# Supplementary material for: Comparative analysis of functional assay evidence use by ClinGen Variant Curation Expert Panels
Source: Genome Med. 2019 Nov 29;11:77. doi: 10.1186/s13073-019-0683-1 (PMC6884856; doi:10.1186/s13073-019-0683-1)
Supplement: Supplementary file 1 — Additional file 1. Summaries of VCEP disease mechanisms and assay specifications. [file 13073_2019_683_MOESM1_ESM.docx]

*CDH1*

The *CDH1* Variant Curation Expert Panel (VCEP) set guidelines for functional studies of the E-cadherin protein encoded by *CDH1* [1]*.* Cadherins are one of the super families of cell adhesion molecules and are a calcium-dependent transmembrane protein. These proteins serve as a link between cells expressing the same type of cadherin on the extracellular membrane and connect to intracellular processes inside the cell, such as the cytoskeleton, through catenin binding to facilitate cell signaling [2]. Cell-cell adhesion is a necessary factor in maintaining tissue organization in the body [3]. The disruption of this process can lead to tumor invasion, the first phase of metastasis [4], which is thought to be caused by the loss of cell junctions and detachment of cells from organized tissue. Loss-of-function variants in the *CDH1* gene are associated with cancer invasiveness through an increase in cell motility [5] and variants in this gene have been linked to hereditary diffuse gastric cancer (HDGC) [6]. Over 150 *CDH1* variants have been identified in patients with HDGC, with about 80% of germline variants encoding a premature stop of translation (nonsense, indel, or splice-site), while the remaining 20% are missense variants [7].

*In vitro* studies commonly test *CDH1* variants for retention of two main functions, cell-cell adhesion and invasion suppression, through an aggregation assay or collagen invasion assay, respectively. Chinese hamster ovary cells (CHO-K1) are often used for these assays because the cells lack endogenous CDH1 [8]. Cell motility assays have shown that variants in the extracellular portion of E-cadherin are more motile than variants in the intracellular domain or cytoplasmic tail [9]. In conjunction with these assays, protein localization, stability, folding, and interactions with binding partners are also frequently assessed with functional assays, as defects in these processes can lead to protein degradation [10]. Missense variants that result in protein destabilization or misfolding are suggested to be highly damaging, as this leads to premature E-cadherin degradation and loss of function [11]. PS3 was approved for application to variants demonstrating abnormal out-of-frame transcripts and PS3_supporting was approved for application to variants with in-frame transcripts [1]. No assays testing the effect of missense variants were approved by the VCEP. However, 20% of patients with HDGC have a missense variant in *CDH1* [7], so while the functional studies assessed by the VCEP appear to be poor predictors of pathogenicity, continual review of these assays and any additional functional assays would be appropriate as more functional data becomes available.

Hearing Loss

Cochlin, encoded by *COCH*, is a secreted protein that localizes intracellularly primarily to the endoplasmic reticulum and Golgi network [12]. It is highly expressed in the cochlea and in cells associated with vestibular nerve fibers, two structures involved in the transmission of sound waves [13–15]. *COCH* dominant negative or gain-of-function variants that alter cochlin folding are associated with autosomal dominant nonsyndromic deafness 9 (DFNA9) due to abnormal protein dimerization, localization, and secretion [12,16,17]. Thus, the VCEP determined functional studies assessing these qualities by western blot or immunofluorescence and showing abnormal patterns can be applied at PS3_moderate, while studies showing no evidence of altered patterns compared to wild type cochlin can be applied at BS3_supporting.

*GJB2* encodes connexin 26, a gap junction protein involved in potassium ion recycling required for inner ear sensory transduction [18]. Loss-of-function and dominant negative *GJB2* variants are associated with autosomal recessive nonsyndromic deafness 1 (DFNB1) [19,20] and autosomal dominant nonsyndromic deafness 3 (DFNB3) [21], respectively. The HL VCEP approved assays measuring gap junction formation and function (electrical coupling and dye transfer) in cDNA-transfected Xenopus oocytes or mammalian cell lines for functional testing of *GJB2* variants, given that proper negative controls (water-injected or non-transfected cells) were used. *GJB2* variants exhibiting a statistically significant reduction in gap junction function compared to wild type were approved by the HL VCEP for PS3_moderate, while variants with no effect were approved for BS3_supporting.

*SLC26A4*, encoding the anion exchange protein pendrin, also supports ion transport in the inner ear [22,23]. Loss-of-function variants in *SLC26A4* are associated with autosomal recessive non-syndromic deafness 4 (DFNB4) [24]. Assays monitoring anion transport, either by radioactive or fluorescent labeling, in Xenopus oocytes or mammalian cell lines were approved by the VCEP if, similar to assays approved for *GJB2*, negative controls were included. A statistically significant reduction in transport, either qualitative or quantitative, compared to wild type can be used as PS3_supporting, while no difference can be used as BS3_supporting. It is important to note that unlike other specifically approved functional assays, evidence from these assays can only be applied at a level of PS3_supporting or BS3_supporting due to their lower positive predictive value, as estimated by the VCEP. Although no specific assays were approved for the remaining genes, “well-validated” functional studies of variants in these six genes can be applied at a PS3/BS3 evidence strength level of supporting if appropriate controls were used.

Inherited Cardiomyopathy

Inherited cardiomyopathies are acquired in an autosomal dominant fashion and, despite heterogeneity in the disease mechanism between different forms, all are associated with heart failure [25]. The *MYH7* protein encodes the β (beta) myosin heavy chain, which is expressed in cardiac muscle and all slow skeletal muscle fibers [26]. This protein is vital for generating the mechanical force needed for muscles to contract [27]. Specifically, human cardiac β myosin is a mechanoenzyme that converts energy from adenosine triphosphate (ATP) hydrolysis to a mechanical force, driving and maintaining muscle contractility of the heart [28]. The VCEP concluded that *in vivo* models that alter normal protein levels, such as transgenic or knock-out mouse models or zebrafish knock-down models, are ineffective as they do not provide information about the effect of a particular variant and are not reflective of the gain-of-function disease mechanism prevalent in cardiomyopathies [29]. No other parameters were set by the VCEP when assessing mammalian models. Furthermore, given the poor predictive value of the sixteen *in vitro* assays evaluated in the VCEP literature review, no *in vitro* assays were approved for PS3/BS3 application at any strength level.

*PAH*

The *PAH* VCEP published functional study guidelines for variants in the phenylalanine hydroxylase (*PAH*) gene associated with the autosomal recessive disorder phenylketonuria (PKU) [30]. PKU is caused by loss-of-function variants in the *PAH* gene that interfere with the tetrahydrobiopterin (BH_4_)-dependent conversion of the amino acid phenylalanine (Phe) to tyrosine (Tyr) by the PAH enzyme. Variants that interfere with normal enzyme activity cause a buildup of phenylalanine in the blood, leading to a neurotoxic effect that can result in intellectual disability [31]. PKU is an actionable disorder and dietary intervention is used to prevent neurotoxic side effects [32]. A subset of PKU patients, most often those with more mild forms of the disease, respond well to treatment with the cofactor BH_4_, which can replace dietary restriction of Phe [33,34]. *In vitro* PAH enzyme activity has been shown to correlate with the severity of the PKU disease [35] and the *PAH* VCEP approved in vitro PAH enzyme activity assays to support PS3/BS3 criteria [30]. Depending on the type and location of the variant, different clinical manifestations can arise with varying levels of severity dependent on the PAH levels. Another factor to consider when evaluating functional evidence is that patients with compound heterozygous genotypes may not reflect the predicted phenotype from averaging the activity level of the two variants. While some *PAH* genotypes are homozygous (one study found 8.2% of 1543 genotypes from the BIOPKU database to be homozygous) [36], most are compound heterozygous with two different variants in the *PAH* gene. The original method of averaging the value of two variants from a functional test to predict the percent activity on the patient is often inaccurate [37]. This phenomenon, termed negative interallelic complementation [38], is caused by interaction between the variants and can result in an enzyme activity that differs from the predicted value. The recent development of a cellular model to account for *PAH* negative interallelic complementation [39] may be a useful functional assessment of enzyme activity in compound heterozygotes.

*PTEN*

The PTEN protein is a tumor suppressor involved in regulation of the phosphatidylinositol 3-kinase (PI3K)/AKT pathway via phosphatidylinositol (3,4,5)-trisphosphate (PIP3) dephosphorylation to prevent unchecked cell survival and proliferation [40,41]. Loss-of-function variants in *PTEN* lead to haploinsufficiency [42] and are associated with the autosomal dominant inheritance pattern observed in *PTEN*-related diseases [43], including hamartoma tumor syndrome. To date, a single gain-of-function *PTEN* variant has been described that alters enzymatic specificity to favor 5-oriented phosphoinositides [44].

Seven general assay classes were approved by the *PTEN* VCEP for use in support of PS3/BS3 criteria. Lipid phosphatase activity assays that directly test catalytic function of PTEN were approved if a catalytically inactive control and three biological replicates were included. To serve as PS3 evidence, the variant must exhibit a reduction in phosphatase activity of over 50% compared to wild type. No alteration of phosphatase activity compared to wild type can serve as evidence for BS3_supporting and, if accompanied by a second assay demonstrating no difference from wild type, BS3 can be applied at a strong evidence level. The VCEP also approved splicing assessment for PS3 or BS3 application. Additional functional assays were approved for PS3 at the reduced evidence strength level of supporting. PTEN normally functions to dephosphorylate PIP3, preventing AKT recruitment and phosphorylation, thus PTEN deficient cells contain elevated levels of PIP3 and phosphorylated AKT (pAKT) [45]. The VCEP approved measurements of pAKT levels as an indirect determinant of PTEN phosphatase activity. Decreased PTEN expression levels have also been correlated with PTEN variant pathogenicity in patient populations [46] and assays assessing PTEN protein levels were approved for PS3_supporting. PTEN localizes to both the cytoplasm and nucleus [47], with its presence in the nucleus being key for tumor suppression [48], thus assays finding aberrant PTEN localization were approved for PS3_supporting. Increased cell migration, proliferation, and invasion are common phenotypes associated with defective PTEN and indicative of tumorigenic potential [49,50] and the VCEP approved assays evaluating these traits for PS3_supporting. Finally, transgenic model organisms displaying phenotypes associated with hamartoma tumor syndrome can also be applied at PS3_supporting.

RASopathy

The proteins encoded by genes linked to RASopathy conditions (*BRAF*, *HRAS*, *KRAS*, *MAP2K1*, *MAP2K2*, *PTPN11*, *RAF1*, *SHOC2*, and *SOS1)* function in the Ras/Raf/MAPK signal transduction pathway to conduct extracellular signals to the nucleus, ultimately resulting in gene expression modulation to regulate diverse cell functions, including cell proliferation, differentiation, and apoptosis [51]. Gain-of-function variants in these genes lead to dysregulation of the pathway and are associated with Noonan syndrome (*PTPN11*, *SHOC2*, *SOS1*, *RAF1*), Cardio-facio-cutaneous syndrome (*BRAF*, *MAP2K1*, *MAP2K2*, *KRAS*), and Costello syndrome (*HRAS*), collectively referred to as the RASopathies [52]. The RASopathy VCEP approved a number of gene-specific assays for PS3/BS3 application; however, unlike some of the other VCEPs, no particular guidance was given for modifying evidence level strength to moderate or supporting. MAP2K1/2 and ERK1/2 are downstream of many genes linked to RASopathy and assays assessing their activation via phosphorylation were approved for PS3/BS3 evidence for variants in these genes. *In vitro* assays showing increased, prolonged, or constitutive phosphorylation compared to wild type can be used for PS3, while no alteration of phosphorylation can be used for BS3. Assays evaluating the catalytic activity of kinases (BRAF, RAF1), phosphatases (PTPN11), and guanine exchange (SOS1) implicated in RASopathy were also approved for PS3 application. Binding assays testing the persistence of protein-protein interactions, specifically HRAS binding to RAF1 (or a truncated version of the protein, RBD) were approved for PS3 application if coprecipitation experiments show increased binding. Finally, assays of the myristoylation status of a specific residue of SHOC2 were approved for PS3. SHOC2 normally localizes to the cytoplasm and nucleus in starved cells, but addition of the fatty acid myristate to an N-terminal glycine is known to restrict SHOC2 to the cell membrane and cause increased MAPK activation [53]. Knock-in models of variants in certain genes associated with RASopathy (*BRAF*, *HRAS*, *PTPN11*, *RAF1*, *SOS1*) were approved in three organisms: mouse, drosophila, and zebrafish and must display a minimum number of defined “deleterious” features for PS3 or BS3 application.

References

1. Lee K, Krempely K, Roberts ME, Anderson MJ, Carneiro F, Chao E, et al. Specifications of the ACMG/AMP variant curation guidelines for the analysis of germline *CDH1* sequence variants. Hum Mutat. 2018 Nov;39(11):1553–68.

2. Takeichi M. Cadherin cell adhesion receptors as a morphogenetic regulator. Science. 1991 Mar 22;251(5000):1451–5.

3. Green KJ, Getsios S, Troyanovsky S, Godsel LM. Intercellular junction assembly, dynamics, and homeostasis. Cold Spring Harb Perspect Biol. 2010 Feb;2(2):a000125.

4. Gabbert H, Wagner R, Moll R, Gerharz CD. Tumor dedifferentiation: an important step in tumor invasion. Clin Exp Metastasis. 1985;3(4):257–79.

5. Birchmeier W, Behrens J. Cadherin expression in carcinomas: role in the formation of cell junctions and the prevention of invasiveness. Biochim Biophys Acta. 1994 May 27;1198(1):11–26.

6. Guilford P, Hopkins J, Harraway J, McLeod M, McLeod N, Harawira P, et al. E-cadherin germline mutations in familial gastric cancer. Nature. 1998 Mar 26;392(6674):402–5.

7. Melo S, Figueiredo J, Fernandes MS, Gonçalves M, Morais-de-Sá E, Sanches JM, et al. Predicting the Functional Impact of CDH1 Missense Mutations in Hereditary Diffuse Gastric Cancer. Int J Mol Sci. 2017 Dec 12;18(12).

8. Suriano G, Oliveira C, Ferreira P, Machado JC, Bordin MC, De Wever O, et al. Identification of CDH1 germline missense mutations associated with functional inactivation of the E-cadherin protein in young gastric cancer probands. Hum Mol Genet. 2003 Mar 1;12(5):575–82.

9. Mateus AR, Simões-Correia J, Figueiredo J, Heindl S, Alves CC, Suriano G, et al. E-cadherin mutations and cell motility: a genotype-phenotype correlation. Exp Cell Res. 2009 May 1;315(8):1393–402.

10. Figueiredo J, Söderberg O, Simões-Correia J, Grannas K, Suriano G, Seruca R. The importance of E-cadherin binding partners to evaluate the pathogenicity of E-cadherin missense mutations associated to HDGC. Eur J Hum Genet. 2013 Mar;21(3):301–9.

11. Simões-Correia J, Figueiredo J, Lopes R, Stricher F, Oliveira C, Serrano L, et al. E-cadherin destabilization accounts for the pathogenicity of missense mutations in hereditary diffuse gastric cancer. PLoS One. 2012;7(3):e33783.

12. Robertson NG, Hamaker SA, Patriub V, Aster JC, Morton CC. Subcellular localisation, secretion, and post-translational processing of normal cochlin, and of mutants causing the sensorineural deafness and vestibular disorder, DFNA9. J Med Genet. 2003 Jul 1;40(7):479–86.

13. Robertson NG, Khetarpal U, Gutiérrez-Espeleta GA, Bieber FR, Morton CC. Isolation of Novel and Known Genes from a Human Fetal Cochlear cDNA Library Using Subtractive Hybridization and Differential Screening. Genomics. 1994 Sep 1;23(1):42–50.

14. Robertson NG, Skvorak AB, Yin Y, Weremowicz S, Johnson KR, Kovatch KA, et al. Mapping and Characterization of a Novel Cochlear Gene in Human and in Mouse: A Positional Candidate Gene for a Deafness Disorder, DFNA9. Genomics. 1997 Dec 15;46(3):345–54.

15. Robertson NG, Lu L, Heller S, Merchant SN, Eavey RD, McKenna M, et al. Mutations in a novel cochlear gene cause DFNA9, a human nonsyndromic deafness with vestibular dysfunction. Nat Genet. 1998 Nov;20(3):299–303.

16. Liepinsh E, Trexler M, Kaikkonen A, Weigelt J, Bányai L, Patthy L, et al. NMR structure of the LCCL domain and implications for DFNA9 deafness disorder. EMBO J. 2001 Oct 1;20(19):5347–53.

17. Yao J, Py BF, Zhu H, Bao J, Yuan J. Role of protein misfolding in DFNA9 hearing loss. J Biol Chem. 2010 May 14;285(20):14909–19.

18. Zhao H-B, Kikuchi T, Ngezahayo A, White TW. Gap junctions and cochlear homeostasis. J Membr Biol. 2006;209(2–3):177–86.

19. Kelsell DP, Dunlop J, Stevens HP, Lench NJ, Liang JN, Parry G, et al. Connexin 26 mutations in hereditary non-syndromic sensorineural deafness. Nature. 1997 May;387(6628):80–3.

20. RamShankar M, Girirajan S, Dagan O, Ravi Shankar HM, Jalvi R, Rangasayee R, et al. Contribution of connexin26 (GJB2) mutations and founder effect to non-syndromic hearing loss in India. J Med Genet. 2003 May 1;40(5):e68.

21. Marziano NK, Casalotti SO, Portelli AE, Becker DL, Forge A. Mutations in the gene for connexin 26 (GJB2) that cause hearing loss have a dominant negative effect on connexin 30. Hum Mol Genet. 2003 Apr 15;12(8):805–12.

22. Scott DA, Karniski LP. Human pendrin expressed in *Xenopus laevis* oocytes mediates chloride/formate exchange. Am J Physiol Physiol. 2000 Jan;278(1):C207–11.

23. Scott DA, Wang R, Kreman TM, Sheffield VC, Karniski LP. The Pendred syndrome gene encodes a chloride-iodide transport protein. Nat Genet. 1999 Apr 1;21(4):440–3.

24. Pera A, Dossena S, Rodighiero S, Gandía M, Bottà G, Meyer G, et al. Functional assessment of allelic variants in the SLC26A4 gene involved in Pendred syndrome and nonsyndromic EVA. Proc Natl Acad Sci U S A. 2008 Nov 25;105(47):18608–13.

25. Richardson P, McKenna W, Bristow M, Maisch B, Mautner B, O’Connell J, et al. Report of the 1995 World Health Organization/International Society and Federation of Cardiology Task Force on the Definition and Classification of cardiomyopathies. Circulation. 1996 Mar 1;93(5):841–2.

26. Richard P, Charron P, Carrier ; Lucie, Ledeuil C, Cheav T, Pichereau C, et al. Hypertrophic Cardiomyopathy Distribution of Disease Genes, Spectrum of Mutations, and Implications for a Molecular Diagnosis Strategy. 2003;

27. Jandreski MA, Sole MJ, Liew C-C. Two different forms of beta myosin heavy chain are expressed in human striated muscle. Vol. 77, Hum Genet. 1987.

28. Sivaramakrishnan S, Ashley E, Leinwand L, Spudich JA. Insights into human beta-cardiac myosin function from single molecule and single cell studies. J Cardiovasc Transl Res. 2009 Dec;2(4):426–40.

29. Kelly MA, Caleshu C, Morales A, Buchan J, Wolf Z, Harrison SM, et al. Adaptation and validation of the ACMG/AMP variant classification framework for MYH7-associated inherited cardiomyopathies: recommendations by ClinGen’s Inherited Cardiomyopathy Expert Panel. Genet Med. 2018 Mar 4;20(3):351–9.

30. Zastrow DB, Baudet H, Shen W, Thomas A, Si Y, Weaver MA, et al. Unique aspects of sequence variant interpretation for inborn errors of metabolism (IEM): The ClinGen IEM Working Group and the Phenylalanine Hydroxylase Gene. Hum Mutat. 2018 Nov 1;39(11):1569–80.

31. Flydal MI, Martinez A. Phenylalanine hydroxylase: function, structure, and regulation. IUBMB Life. 2013 Apr 1;65(4):341–9.

32. Bickel H, Gerrard J, Hickmans EM. Influence of phenylalanine intake on phenylketonuria. Lancet (London, England). 1953 Oct 17;265(6790):812–3.

33. Fiege B, Blau N. Assessment of tetrahydrobiopterin (BH4) responsiveness in phenylketonuria. J Pediatr. 2007 Jun;150(6):627–30.

34. Kure S, Hou DC, Ohura T, Iwamoto H, Suzuki S, Sugiyama N, et al. Tetrahydrobiopterin-responsive phenylalanine hydroxylase deficiency. J Pediatr. 1999 Sep;135(3):375–8.

35. Himmelreich N, Shen N, Okun JG, Thiel C, Hoffmann GF, Blau N. Relationship between genotype, phenylalanine hydroxylase expression and in vitro activity and metabolic phenotype in phenylketonuria. Mol Genet Metab. 2018 Sep;125(1–2):86–95.

36. Wettstein S, Underhaug J, Perez B, Marsden BD, Yue WW, Martinez A, et al. Linking genotypes database with locus-specific database and genotype-phenotype correlation in phenylketonuria. Eur J Hum Genet. 2015 Mar;23(3):302–9.

37. Leandro J, Nascimento C, de Almeida IT, Leandro P. Co-expression of different subunits of human phenylalanine hydroxylase: evidence of negative interallelic complementation. Biochim Biophys Acta. 2006 May;1762(5):544–50.

38. Kaufman S, Max EE, Kang ES. Phenylalanine hydroxylase activity in liver biopsies from hyperphenylalaninemia heterozygotes: deviation from proportionality with gene dosage. Pediatr Res. 1975 Aug 1;9(8):632–4.

39. Danecka MK, Woidy M, Zschocke J, Feillet F, Muntau AC, Gersting SW. Mapping the functional landscape of frequent phenylalanine hydroxylase (PAH) genotypes promotes personalised medicine in phenylketonuria. J Med Genet. 2015 Mar 1;52(3):175–85.

40. Maehama T, Dixon JE. The tumor suppressor, PTEN/MMAC1, dephosphorylates the lipid second messenger, phosphatidylinositol 3,4,5-trisphosphate. J Biol Chem. 1998 May 29;273(22):13375–8.

41. Stambolic V, Suzuki A, de la Pompa JL, Brothers GM, Mirtsos C, Sasaki T, et al. Negative regulation of PKB/Akt-dependent cell survival by the tumor suppressor PTEN. Cell. 1998 Oct 2;95(1):29–39.

42. Kwabi-Addo B, Giri D, Schmidt K, Podsypanina K, Parsons R, Greenberg N, et al. Haploinsufficiency of the Pten tumor suppressor gene promotes prostate cancer progression. Proc Natl Acad Sci. 2001 Sep 25;98(20):11563–8.

43. Eng C. PTEN: One Gene, Many Syndromes. Hum Mutat. 2003 Sep;22(3):183–98.

44. Costa HA, Leitner MG, Sos ML, Mavrantoni A, Rychkova A, Johnson JR, et al. Discovery and functional characterization of a neomorphic PTEN mutation. Proc Natl Acad Sci U S A. 2015 Nov 10;112(45):13976–81.

45. Sun H, Lesche R, Li DM, Liliental J, Zhang H, Gao J, et al. PTEN modulates cell cycle progression and cell survival by regulating phosphatidylinositol 3,4,5,-trisphosphate and Akt/protein kinase B signaling pathway. Proc Natl Acad Sci U S A. 1999 May 25;96(11):6199–204.

46. Tan M-H, Mester J, Peterson C, Yang Y, Chen J-L, Rybicki LA, et al. A Clinical Scoring System for Selection of Patients for PTEN Mutation Testing Is Proposed on the Basis of a Prospective Study of 3042 Probands. Am J Hum Genet. 2011 Jan 7;88(1):42–56.

47. Ginn-Pease ME, Eng C. Increased nuclear phosphatase and tensin homologue deleted on chromosome 10 is associated with G0-G1 in MCF-7 cells. Cancer Res. 2003 Jan 15;63(2):282–6.

48. Gil A, Rodríguez-Escudero I, Stumpf M, Molina M, Cid VJ, Pulido R. A functional dissection of PTEN N-terminus: implications in PTEN subcellular targeting and tumor suppressor activity. Uversky VN, editor. PLoS One. 2015 Apr 15;10(4):e0119287.

49. Tamura M, Gu J, Matsumoto K, Aota S, Parsons R, Yamada KM. Inhibition of cell migration, spreading, and focal adhesions by tumor suppressor PTEN. Science. 1998 Jun 5;280(5369):1614–7.

50. Tamura M, Gu J, Takino T, Yamada KM. Tumor suppressor PTEN inhibition of cell invasion, migration, and growth: differential involvement of focal adhesion kinase and p130Cas. Cancer Res. 1999 Jan 15;59(2):442–9.

51. Cargnello M, Roux PP. Activation and function of the MAPKs and their substrates, the MAPK-activated protein kinases. Microbiol Mol Biol Rev. 2011 Mar 1;75(1):50–83.

52. Rauen KA. The RASopathies. Annu Rev Genomics Hum Genet. 2013 Aug 31;14(1):355–69.

53. Cordeddu V, Di Schiavi E, Pennacchio LA, Ma’ayan A, Sarkozy A, Fodale V, et al. Mutation of SHOC2 promotes aberrant protein N-myristoylation and causes Noonan-like syndrome with loose anagen hair. Nat Genet. 2009 Sep 16;41(9):1022–6.
